# Bringing cellular clarity to the cortical component of ALS with a high‐density multi‐electrode array system

**DOI:** 10.1111/febs.70552

**Published:** 2026-04-21

**Authors:** Zehra Yagmur Erol, Christopher Quintanilla, P. Hande Ozdinler

**Affiliations:** ^1^ Department of Neurology, Feinberg School of Medicine Northwestern University Chicago IL USA; ^2^ Les Turner ALS Center Northwestern University, Feinberg School of Medicine Chicago IL USA; ^3^ Chemistry of Life Processes Institute Northwestern University Evanston IL USA; ^4^ Mesulam Institute for Cognitive Neurology and Alzheimer's Disease Northwestern University, Feinberg School of Medicine Chicago IL USA

**Keywords:** HD‐MEA technology, network dynamics, neurodegeneration, single‐cell electrophysiology, upper motor neurons

## Abstract

There is selective vulnerability in diseases, requiring the understanding of cell‐type specific aspects of neurodegeneration with cellular resolution. Single‐cell electrophysiology enables direct investigation of neuronal excitability, ion‐channel dynamics, and synaptic transmission with high temporal precision, which is crucial in understanding neuronal circuitries and how they are affected in diseases. Microelectrode arrays (MEAs) have emerged as powerful platforms enabling long‐term, parallel, and non‐invasive extracellular measurements. The advent of complementary‐metal‐oxide‐semiconductor (CMOS)‐based and high‐density microelectrode arrays (HD‐MEAs) has expanded the spatial and temporal resolution attainable both in vitro and ex vivo. Combined with acute slices, novel cell‐culture approaches and three‐dimensional (3D) brain organoids, these tools now expedite translational research in disease modeling, neurotoxicity, and pharmacology. Here, we summarise the significance of single‐cell electrophysiology, the advantages of the MEA systems, and the latest biomedical and technological advances in this area of research.

## Graphical abstract

High density multi‐electrode (HD‐MEA) systems enable detailed investigation of healthy and diseased brains in 2D and 3D. Here, we summarise the significance of single‐cell electrophysiology, advantages of the MEA systems with high spatio‐temporal resolution and a large number of recording sites, enabling complex network analysis.

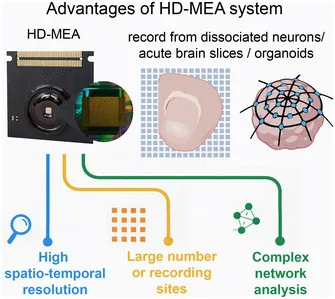

**Abbreviations**
2‐D: two‐dimensional
3‐D: three‐dimensional
ALS: amyotrophic lateral sclerosis
CMOS: complementary metal‐oxide‐semiconductor
HD‐MEA: high‐density microelectrode array
HSP: heredity spastic paraplegia
iPSC: induced pluripotent stem cell
MEA: microelectrode array
PLS: primary lateral sclerosis
SNR: signal‐to‐noise ratio
UMN: upper motor neuron

## Introduction

Neurodegenerative diseases are characterised by selective vulnerability of distinct neuron populations, leading to progressive neurodegeneration and circuit dysfunction [1, 2]. In this Viewpoint, we focus primarily on amyotrophic lateral sclerosis (ALS), a disease of the motor neuron circuitry [3]. ALS has both cortical and spinal cord components, and both the upper and lower motor neurons display progressive degeneration [3, 4, 5]. Although the spinal motor neurons are better studied, understanding the underlying causes of circuit‐level dysfunction in the ALS brain requires more precise tools and applications.

Both clinical and experimental evidence indicates an early phase of cortical hyperexcitability followed by reduced neuronal responsiveness as the disease progresses in ALS [5, 6, 7, 8, 9, 10]. Hyperexcitability has been detected in patients using transcranial magnetic stimulation and is considered an early physiological biomarker for the disease [6, 11, 12]. Sustained experimental induction of cortical hyperactivity in healthy mice results in the development of sporadic ALS‐like features, strongly supporting the link between cortical hyperexcitation and initiation of disease pathology in ALS [13]. One potential explanation could be the alterations in intrinsic and synaptic properties of upper motor neurons (UMNs) at presymptomatic stages, as reported in ALS mouse models [14, 15]. In addition, prominent apical dendrite degeneration detected in the Betz cells of both sporadic and familial cases of ALS, and the UMNs of well‐defined mouse models could contribute to cortical circuit dysfunction in the disease [16]. Understanding the underlying causes of early cortical dysfunction in ALS is an important quest and we need to bring cellular resolution to our investigation.

High‐resolution electrophysiological approaches have been instrumental in defining the cellular basis of neuronal excitability. Intracellular techniques such as whole‐cell and cell‐attached patch‐clamp recordings provide direct access to membrane potential and enable quantification of subthreshold synaptic events, ionic conductance, and intrinsic excitability properties [17, 18]. In addition, complementary functional imaging strategies, including calcium imaging and genetically encoded or dye‐based voltage indicators, enable monitoring of neuronal activity across populations with cell‐type specificity, even though their effective temporal resolution is influenced by indicator kinetics and signal dynamics [19, 20, 21].

While intracellular recordings provide unparalleled access to membrane dynamics, and optical approaches enable population‐level imaging with cell‐type specific resolution, both modalities present inherent trade‐offs in throughput, temporal precision, or scalability across extended neuronal assemblies. Neurodegenerative diseases such as ALS are increasingly recognised as disorders of circuit dysfunction rather than isolated single‐neuron failure, with alterations in synchronisation, propagation, recruitment and excitability emerging at the network level before prominent neuron loss [5, 14, 22]. Addressing these multiscale alterations therefore requires experimental platforms capable of capturing distributed population activity with high temporal precision while remaining compatible with long‐term and large‐scale recordings.

MEA systems contribute to our understanding of integrative neuronal framework by enabling simultaneous extracellular recordings from multiple neurons and spatially distributed regions, thereby linking single‐unit firing with emergent network dynamics [23]. Over several decades, advances in microfabrication and electronics have progressively expanded the spatial resolution and scalability of MEA platforms, setting the stage for the development of modern high‐density configurations [24, 25, 26, 27].

## History and technological evolution of MEA

The concept of parallel extracellular recordings using planar microelectrode arrays emerged in the late 1970s, when early thin‐film electrode arrays were developed to monitor activity from cultured neuronal networks [28, 29]. These initial systems incorporated a limited number of metal electrodes fabricated on glass substrates and enabled long‐term extracellular recordings from dissociated neuronal cultures, establishing the foundation for network‐level electrophysiology [23, 28, 29]. During the 1980s and 1990s, continued advances in microfabrication and thin‐film processing improved electrode stability, signal reproducibility, and compatibility with acute brain slice preparations, facilitating broader adoption of planar MEAs in in vitro and ex vivo studies [29, 30]. Conventional planar MEAs were typically characterised by relatively low channel counts, wider electrode spacing, and reliance on external amplification architectures [23].

A major technological transition occurred with the integration of complementary metal‐oxide‐semiconductor (CMOS) technology into MEA architectures [25, 26]. In contrast to passive arrays, CMOS‐based platforms integrate local amplification circuitry directly beneath or adjacent to individual electrode sites. By amplifying extracellular signals at their source, on‐chip amplification reduces signal degradation associated with long interconnects, minimises parasitic capacitance, and improves signal‐to‐noise ratio [25, 31]. In addition, multiplexed readout architectures reduce wiring constraints by allowing large numbers of electrodes to share readout channels. This architectural strategy enables substantially higher electrode densities and smaller inter‐electrode spacing without requiring proportional increases in external connections [26, 31]. As a result, spatial sampling of extracellular signals can be markedly increased, supporting more precise localisation and distributed detection of neuronal activity across extended network areas [31].

Recent developments facilitated the emergence of high‐density MEAs (HD‐MEAs), characterised by thousands to hundreds of thousands of closely spaced electrodes with micrometer‐scale pitch [24, 32, 33]. More recent large‐area implementations have extended electrode counts beyond 200,000 recording sites, reflecting continued advances in electronics integration and scalability [24, 33]. Overall, the transition from early planar arrays to CMOS‐based HD‐MEAs represents incremental progress in integration density, signal conditioning, and data handling, while maintaining the established principle of extracellular voltage recording.

The transition from early planar arrays to CMOS‐based HD‐MEAs reflects incremental advances in microfabrication, integration density, and signal processing. While the fundamental approach‐recording extracellular voltage fluctuations generated by neuronal activity remains consistent with earlier MEA systems, improvements in electronics integration and data acquisition have substantially expanded the spatial scale and analytical resolution of these recordings [23, 24, 31, 32].

## Advantage of the HD‐MEA technology

Single‐cell electrophysiology remains the gold standard for measuring neuronal electrical activity at the cellular level, providing direct access to membrane potential dynamics and synaptic mechanisms. However, intracellular approaches require membrane access and are inherently limited in throughput and scalability across extended neuronal populations.

Optical techniques, including calcium imaging and genetically encoded voltage indicators, enable population‐level activity mapping with cell‐type specificity. These approaches offer powerful spatial visualisation of activity patterns but depend on fluorescent reporters whose kinetics and optical constraints influence temporal precision and quantitative voltage interpretation [19, 20]. The HD‐MEAs complement these modalities by enabling simultaneous extracellular recordings from thousands of sites while preserving cellular and tissue integrity (Fig. 1A). Because MEAs do not require membrane rupture or optical excitation, they provide a minimally invasive, label‐free platform that enables long‐term monitoring of network activity in dissociated cultures, organotypic slices, or brain organoids [23, 31] (Fig. 1B,C). Three‐dimensional (3D) neuronal models, including brain organoids, provide increased structural and cellular complexity compared to two‐dimensional cultures and mimic many aspects of cortical neuron differentiation and development [34, 35].

**Fig. 1:**
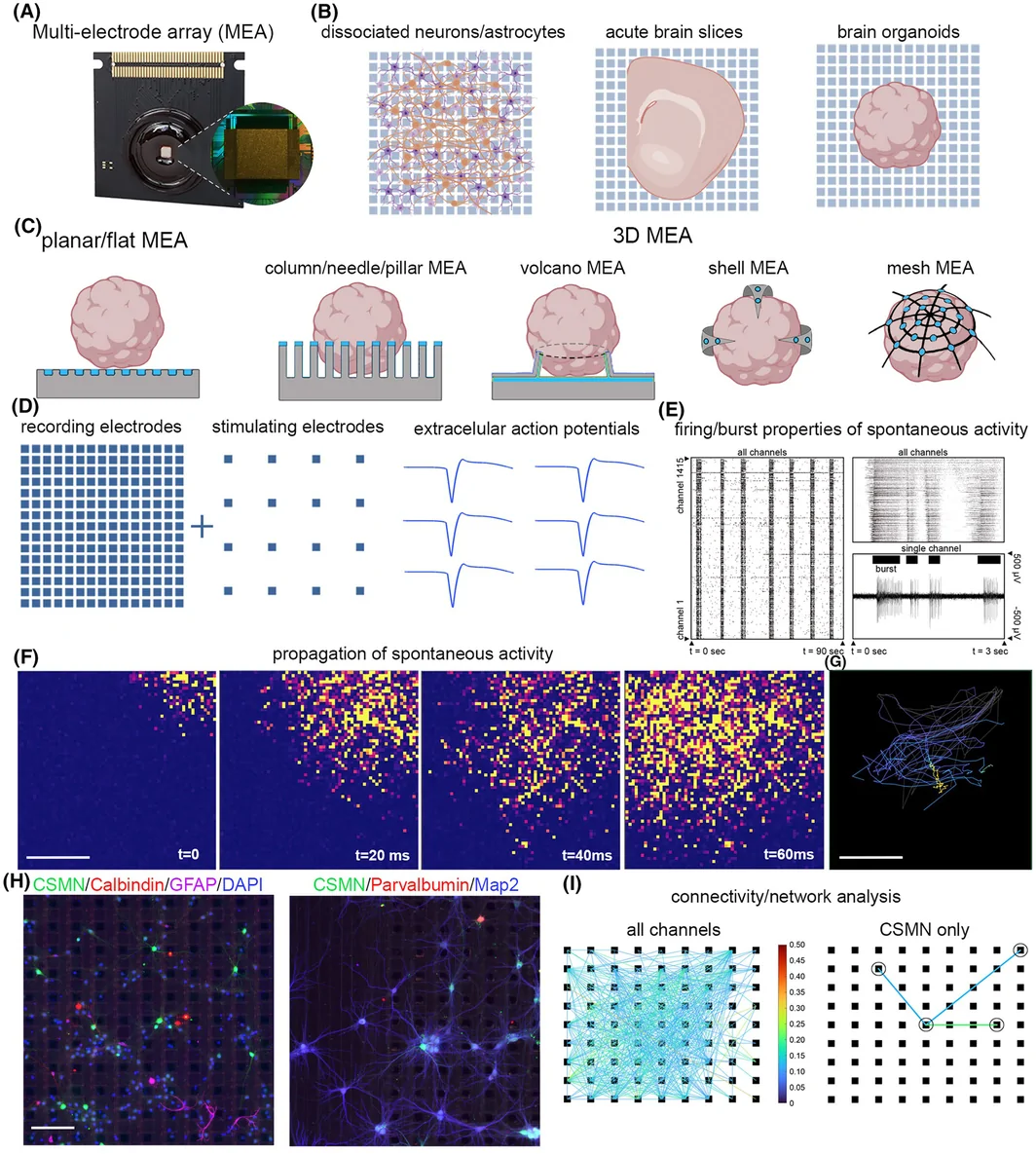
HD‐MEA enables multiscale extracellular electrophysiological recordings across 2D and 3D neural preparations, integrating targeted stimulation, propagation analysis and network‐level activity. (A) Representative image of a MEA chip. The active area is enlarged on the right. (B) Dissociated cells, acute slices or organoids can be studied with MEA. (C) Comparison of planar and three‐dimensional (3D) MEA configurations. Planar/flat arrays interface with cells at the surface, whereas 3D architectures (column/needle/pillar, volcano‐shaped, shell, and mesh MEAs) extend electrodes into tissue, improving cell‐electrode coupling and signal acquisition in thick preparations. (D) HD‐MEAs have many active channels, and some can induce local stimulation, resulting in many waveforms and action potentials that simultaneously become active. (E) Spontaneous activity as well as firing and burst activities can be studied for each active channel over time. (F) Propagation of spontaneous activity of cultured cortical neurons across the HD‐MEA system at four representative time points. Color coding reflects activity intensity, including baseline (blue), and representing increasing levels of neuronal firing (yellow and pink). This visualisation illustrates the spatiotemporal spread of network events across the array. Scale bar: 1200 μm. (G) Summary of the trajectory for each network event including onset (gray), middle (blue) and late (yellow) phase across the HD‐MEA (example from a 3 min recording). Scale bar: 1200 μm. (H) HD‐MEA coupled with immunocytochemistry and imaging bring cellular clarity to electrophysiological assessments Scale bar: 120 μm (equivalent to two electrode pitches, corresponding to center‐to‐center distance across three electrodes). (I) Connectivity network analyses reveal the network dynamics both at a group and single cell level.

The HD‐MEA recordings bridge single‐neuron firing with population‐level oscillations, clarifying how cellular activity integrates within functional microcircuits. Recent complementary CMOS‐based platforms integrate thousands of recording and stimulation sites directly on silicon substrates, enabling dense spatial sampling, improved signal‐to‐noise ratio through on‐chip amplification [23, 31, 36]. For example, commonly used HD‐MEA systems incorporate 4096 microelectrodes with 42–80 μm pitch and active sensor areas up to 5 mm^2^, supporting simultaneous analysis of single active units and large‐scale network connectivity on the same platform (Fig. 1D). Continued advances in integration density have expanded electrode counts to 26,400 [32] and in recent large‐scale implementations, up to 236,880 recording sites [33, 37].

In addition, HD‐MEAs enable integration of functional electrophysiology with structural and molecular analyses. In recent cell culture studies using 3Brain HD‐MEA platforms, extracellular network metrics, including firing rate distributions, burst structure, inter‐burst intervals, and spatial synchronisation were quantitatively aligned with post hoc immunocytochemical markers of cellular phenotype [38]. Such combined functional and structural approaches allow investigation of how specific cellular alterations translate into measurable network‐level signatures. Single‐cell recordings obtained using MEAs capture action potentials and local field potentials with millisecond temporal resolution, providing the foundation for understanding neuronal excitability, plasticity‐related dynamics and functional connectivity (Fig. 1E) [23, 31]. Beyond cellular‐level measurements, MEA systems enable investigation of coordinated network activity across spatially distributed neuronal populations [23, 27] (Fig. 1F,G). When coupled with immunocytochemistry and high‐resolution imaging, electrophysiological recordings can be spatially aligned with identified cellular phenotypes present within active channels, enabling correlation of functional activity with defined neuronal and neuron‐nonneuronal cell interaction [39] (Fig. 1H,I).

One of the key features of HD‐MEAs is their ability to deliver controlled electrical stimulation through selected microelectrodes while simultaneously recording neuronal activity (Fig. 1D). This capability is particularly important to assess neuronal connectivity, network formation, and neuronal responses to external stimulation, enabling investigation of closed‐loop network formation and modulation, especially on acute slices preparations [24, 31]. This feature also provides a framework for probing microcircuit and network‐level responsiveness with high spatial and temporal resolution.

Neuronal networks generate rich spatiotemporal patterns of activity in which action potentials propagate across interconnected microcircuits with characteristic timing, direction, and recruitment dynamics [27, 31]. Disruptions in propagation velocity, synchrony, and microcircuit recruitment are frequently associated with neurodegenerative conditions. The dense spatial sampling provided by HD‐MEAs enables mapping of spike propagation trajectories, initiation zones, synchronisation patterns, and population recruitment across extended network areas [27, 31, 40] (Fig. 1F). When combined with targeted electrical stimulation, these platforms further support causal assessment of activity flow and microcircuit responsiveness [31]. Studying these propagation signatures under physiological and pathological conditions provides insight into selective vulnerability, axonal and synaptic dysfunction, and circuit‐level breakdown (Fig. 1F,G). These high‐content electrophysiological readouts also create a powerful framework for translational applications, including novel drug‐discovery platforms, where improved propagation dynamics and restored functional connectivity serve as a quantitative outcome measure for preclinical drug discovery efforts and for the identification of target‐engagement biomarkers for therapeutic interventions [6, 23, 27, 41].

## Challenges of using HD‐MEA

Despite their many advantages, the HD‐MEA present several limitations. One major limitation is their inability to directly measure membrane potential with accuracy, as extracellular recordings do not provide access to resting membrane potential or the subthreshold synaptic activity. Some of these limitations can be addressed with nanoelectrode arrays allowing for intracellular recordings, which overcome limitations of detecting subthreshold signals [42, 43]. Likewise, similar limitations bring confounding variables to connectivity mapping studies, such that it is not always clear which neurons are functionally connected in the planar HD‐MEAs. The nanoelectrodes are shown to be useful in this regard [44].

The second limitation is the difficulty in reliably detecting signals from subcellular compartments. Microelectrode‐based HD‐MEAs combined with sparse culture conditions have been shown to capture signals originating from axonal arbours [31], allowing the analysis of action potential propagation along individual axons [27, 40].

The third limitation is that extracellular signals are usually made up of multiple neurons in proximity to a specific electrode. Spike‐sorting algorithms such as SpyKing, HDSort, HerdingSpikes2, and Kilosort2 rely on different parameters to determine single‐unit versus multi‐unit activity [45, 46, 47, 48]. Achieving an optimal balance between accuracy and sensitivity remains a major hurdle [49]. Tools such as SpikeInterface allow for comparing multiple spike sorters on the same data and demonstrate the potential of consensus when sorting through multiple spike sorters to standardise high density array studies [49].

The fourth limitation of HD‐MEAs is the efficacy of signal detection in complex and thick tissues. The development of 3D‐MEA architectures, including pillar, needle, and volcano‐shaped microelectrodes increases tissue penetration and significantly improves signal‐to‐noise ratio (SNR). In thick preparations such as acute brain slices and brain organoids, neurons often reside hundreds of micrometers above the electrode surface, causing rapid signal attenuation and increased background noise. Simultaneous signal detection from superficial and deep layers of acute brain slices is also limited without penetrating electrodes. Development of penetrating micro and nanopillar electrode arrays can enhance recording from thick tissue [50]. For complex organoid tissues, self‐folding arrays can be used, providing a simultaneous signal from multiple sites without invasive penetrating electrodes [51, 52]. Alternatively stretchable mesh arrays also enable non‐invasive recordings of organoid tissue [53, 54, 55] (Fig. 1C).

## HD‐MEA technology in drug discovery

Since non‐neuronal cells contribute to the health and function of neurons [56, 57, 58], it is important to investigate how their dynamic interaction impact neuronal health and connectivity within the context of disease. Amyotrophic lateral sclerosis (ALS) is a good example of such diseases, because astrocytes and microglia are implicated in motor neuron degeneration, and diseased non‐neuronal cells contribute to disease pathology [59]. Riluzole, the first FDA‐approved therapy for ALS, is thought to act primarily on astrocytes rather than directly on motor neurons [60]. It reduces the extent of astrogliosis and mitigates the harmful effect of gliosis on vulnerable neurons. Therefore, it is important to assess not only the neurons but also the non‐neuronal cells, and their contributions to neuronal degeneration. HD‐MEA platforms provide a scalable framework for assessing network‐level consequences of such multicellular interactions, enabling parallel monitoring of neuronal activity within heterogeneous cellular environments [23]. Importantly, HD‐MEAs do not directly measure the function of non‐neuronal cells; rather, they capture the network‐level effects of neuron–glia interactions, allowing the contribution of astrocytes to be inferred indirectly [23].

Preclinical testing for compounds is of great interest, as it is essential to identify compounds that improve the health and function of diseased neurons. Since the MEA system brings cellular resolution to detailed electrophysiological assessments, single‐cell and network‐level responses to pharmacological compounds reveal many levels of information relevant to compound screening. In line with these assessments, the multi‐well HD‐MEAs are developed with formats permitting simultaneous functional assays of multiple preparations. They are exceptionally versatile for parallel testing of drug libraries, with output metrics such as mean firing rate, burst index and synchrony providing robust quantitative endpoints [61]. Multi‐well HD‐MEA have thus proven suitable for neurotoxicity screenings and pharmacological profiling in vitro, with outcomes aligning closely with in vivo models [41].

Additional level of translational relevance is achieved when induced pluripotent stem cell (iPSC)‐derived neurons are integrated with HD‐MEA platforms [39]. Patient‐derived iPSC neurons enable functional assessment of disease‐relevant electrophysiological phenotypes in a human cell context [7]. Because many neurodegenerative disorders exhibit heterogeneous etiologies, utilising patient‐derived neuronal networks allows evaluation of pharmacological responses in systems that reflect individual genetic backgrounds. iPSC‐derived neuronal networks on HD‐MEAs have been reported to recapitulate disease‐associated hyperexcitability and network abnormalities observed in conditions such as ALS, epilepsy, and autism [7, 39, 41]. In addition, brain organoids, generated from cells obtained from patients are exceptionally valuable in building a platform that informs a cortical neuron response to compound treatment. This opens the door for personalised medicine approach, with the quest of finding the compounds that the patients' neurons respond to in the most effective way, and eliminating the compounds from their potential treatment regimen, if their differentiated cells/neurons do not respond to treatment. This approach may also improve inclusion criteria for certain drug treatment regimens that are currently in clinical trials and help determine patients who would best respond to treatment.

One of the potential limitations of using iPSC could be the inability of generating the neurons of interest with directed differentiation protocols. One such neuron population are the upper motor neurons (UMNs) that reside in deep layers of the motor cortex and are responsible for the initiation and modulation of voluntary movement. The UMNs degenerate in diseases such as ALS, hereditary spastic paraplegia (HSP) and primary lateral sclerosis (PLS) [5]. iPSC differentiation protocols generate spinal and cortical neurons at large but directed differentiation into distinct neuron types is very challenging, and effective generation of UMNs has not yet been reliably achieved. iPSCs‐based approaches are highly valuable for assessing neuronal toxicity, and treatment responses in a human cellular context. However, more complex systems may be required to fully recapitulate the cellular diversity and structural complexity of the human brain.

## Conclusion and future perspectives

Single‐cell electrophysiology, optical imaging approaches and HD‐MEA technologies collectively contribute to modern functional neurobiology. Intracellular recordings provide mechanistic insight into membrane and synaptic processes, while optical methods enable cell‐type‐specific activity mapping across neuronal populations. HD‐MEAs complement these modalities by capturing large‐scale extracellular network dynamics with high temporal resolution and scalable parallelisation. The evolution from passive planar arrays to bidirectional CMOS‐based HD‐MEAs reflects advances in microfabrication, electronics integration, and computational analysis rather than the emergence of a fundamentally new recording principle. A transcriptomic and spatially resolved molecular atlas of the adult mouse brain [62, 63] along with atlases of the developing [64, 65] and ageing [66] brain are creating new understanding of the degree of heterogeneity within a given class of neuron. Building on this insight, functional connectome databases [67, 68] are making it possible to translate cell‐type specific alterations to a better understanding of circuit level impact. The inhibitory circuit of the motor cortex has been implicated in recent postmortem and clinical studies in ALS. Existing atlases of the mouse motor cortex map [69, 70] has created an important foundation on which to build cell‐based electrophysiology insight, especially in relation to dynamic excitability alterations in ALS. Incorporating HD‐MEA and cell‐based temporal characterisations of vulnerable excitatory and inhibitory neurons will be an important addition to drug discovery efforts for degenerative disease, paving the way for effective and personalised therapies.

The technology continues to evolve toward smaller feature sizes (i.e. 0.35–0.18 μm nodes), reduced heat generation, and enhanced on‐chip digitisation, paving the way for real‐time feedback and multimodal integration. Novel designs enable closed‐loop, depth‐resolved functional phenotyping of iPSC‐derived culture lines and brain organoids, an emerging bridge between cellular physiology and translational neuropharmacology [31, 41]. Many novel HD‐MEA platforms exemplify this trend, merging high‐throughput electrophysiology with 3D tissue compatibility and programmable stimulation.

The convergence of advanced computational analytics, including machine‐learning‐based multi‐level deep analyses, multi‐modal sensing, and adaptive stimulation is expected to redefine in vitro as well as ex vivo studies in the field of neuroscience, turning HD‐MEAs into indispensable tools for both mechanistic discovery and preclinical drug screening. These recent and rapid advances on HD‐MEA technology support fundamental and translational neuroscience, offering unprecedented scalability, resolution, and relevance for modeling, understanding and developing effective treatments for the complex human brain disorders. When HD‐MEAs are combined with the more traditional electrophysiological recording paradigms, it is possible that many of the unanswered questions in neuroscience will be resolved, disease mechanisms will be better understood, and more effective drugs will be identified for complex neurodegenerative diseases.

## Conflict of interest

The authors declare no conflict of interest.

## Author contributions

ZYE performed the literature analysis and wrote the first draft of the manuscript. CQ contributed to the writing of the manuscript and assisted with revisions. PHO supervised the study and revised the manuscript. All authors reviewed and approved the final version of the manuscript.
